# Understanding the health and wellbeing challenges of the food banking system: A qualitative study of food bank users, providers and referrers in London

**DOI:** 10.1016/j.socscimed.2018.05.030

**Published:** 2018-08

**Authors:** C. Thompson, D. Smith, S. Cummins

**Affiliations:** a Department of Social and Environmental Health Research, London School of Hygiene and Tropical Medicine, 15-17 Tavistock Place, London WC1H 9SH, United Kingdom; b Department of Geography and Environment, University of Southampton, University Road, Southampton, SO17 1BJ, United Kingdom

**Keywords:** Food poverty, London, Qualitative, Health inequalities, Food banks, Diet

## Abstract

In the UK, food poverty has been associated with conditions such as obesity, malnutrition, hypertension, iron deficiency, and impaired liver function. Food banks, the primary response to food poverty on the ground, typically rely on community referral and distribution systems that involve health and social care professionals and local authority public health teams. The perspectives of these key stakeholders remain underexplored. This paper reports on a qualitative study of the health and wellbeing challenges of food poverty and food banking in London. An ethnographic investigation of food bank staff and users was carried out alongside a series of healthcare stakeholder interviews. A total of 42 participants were interviewed. A Critical Grounded Theory (CGT) analysis revealed that contemporary lived experiences of food poverty are embedded within and symptomatic of extreme marginalisation, which in turn impacts upon health. Specifically, food poverty was conceptualised by participants to: firstly, be a barrier to providing adequate care and nutrition for young children; secondly, be exacerbated by lack of access to adequate fresh food, food storage and cooking facilities; and thirdly, amplify existing health and social problems. Further investigation of the local government structures and professional roles that both rely upon and serve to further embed the food banking system is necessary in order to understand the politics of changing welfare landscapes.

## Highlights

• Food poverty both exacerbates existing health problems and cause new ones.
• Healthcare professionals prioritise different concerns than food bank users.
• Material deprivation amplifies food poverty via lack of household food facilities.

## 1 Introduction

### 1.1 Food poverty, inequality and health

Food poverty, or household food insecurity, is a social determinant of health (Raphael, 2009). Over the last decade it has become increasingly relevant to UK public health due the continuing retreat of the welfare state, increasing inequality and the impact of sustained public sector austerity stemming from the 2007 economic recession (Ashton et al., 2014). A growing body of literature, predominantly from North America, has observed negative associations between food poverty and health outcomes (Gitterman et al., 2015). In high-income nations, food poverty contributes, paradoxically, to both malnutrition and obesity. Poorer households find themselves unable to afford enough food (Griffith et al., 2013), and the food that they can afford is often poor quality, energy dense and low in nutrients (Dinour et al., 2007). Such diets are associated with a range of conditions including hypertension, iron deficiency, and impaired liver function (Dinour et al., 2007; Markovic and Natoli, 2009). This paper explores how the health and wellbeing challenges associated with food poverty are perceived by both those experiencing them and the health and social care professionals who treat them.

The term food poverty is typically used interchangeably with ‘food insecurity’ (as opposed to food security) (Dowler and O'Connor, 2012; Pinstrup-Andersen, 2009). Although originally used to characterise the nutritional status of nations, it is now widely used to refer to broader problems related to household food status. In this context, food poverty/insecurity is defined as: limited or uncertain availability of nutritionally adequate and safe foods or limited or uncertain ability to acquire acceptable foods in socially acceptable ways (Taylor and Loopstra, 2016). Locating the issue within a rights-based approach recognises the clear interdependence between the (human) right to food and the right to health (Dowler and O'Connor, 2012). Food poverty has varying degrees of severity ranging from worry about whether there will be enough food through to compromising quality and quantity, and then going without food and experiencing hunger (Taylor and Loopstra, 2016). Despite the scale of the problem, there is still no national surveillance system in the UK to monitor food insecurity, as there are in the US and Canada (Loopstra et al., 2015; Smith et al., 2018). UNICEF estimates that in the UK, 19% of all children under the age of 15 live with someone who is at least moderately food insecure, making the UK one of the worst performing nations in the European Union (Pereira et al., 2017). A reliance on estimates and a lack of national-level measurements serve to undermine calls for a national-level response to this emergent public health crisis. This paper contributes to the growing literature on food banking and the evidence base on the health implications of food poverty by exploring the perspectives of key stakeholders in the UK food banking system.

### 1.2 Advanced marginality

The setting for this study is Greater London, where inequality is high, food poverty is a growing problem and increases in the provision of food banking services have been the most rapid (CPAG, 2012; London Assembly, 2013). Given this context, we utilise Wacquant's (2007) notion of ‘advanced marginality’ as a theoretical framing of (food) poverty. This entails situating lived experiences of food poverty within the mechanisms of contemporary urban poverty. Specifically, how marginalized communities become physically, geographically and economically disconnected from wider society via structural barriers to full citizenship. The statecraft of neo-liberal governance has resulted in the growth of social insecurity through the pathways of both insecure working conditions and punitive welfare regimes (Cummins, 2016).

Welfare policy is a structural barrier because it is increasingly informed by a behaviourist philosophy relying on deterrence, surveillance, stigma, and graduated sanctions to modify conduct (Wacquant, 2009). In the UK, food banks are opening in areas experiencing greater cuts in local spending and higher rates of welfare benefit sanctions (Loopstra et al., 2015). Benefit sanctions, the stoppage of payments to recipients on the grounds of alleged non-compliance, have been identified as a major pathway into (food) poverty (Adler, 2016). Detailed analysis of Trussell Trust data in the UK (the country's largest food banking organisation) shows that the main reason for visiting food banks, including repeat visits by households, is due to benefit changes and delays (Garratt, 2017).

The links between insecure working conditions and (food) poverty are less clear. Although it has been identified by food aid organisations and campaigners as a topic the UK Government urgently needs to explore (Cooper et al., 2014). The changing nature of work through increasing use of zero-hours-contracts, whereby employees are guaranteed no set amount of work at all over a weekly or monthly basis (despite being required to be constantly available for work) has caused widespread concern. Most notably about the insecurity, benefit-claiming complications, poverty, and reliance on services including food banks and pay-day-loan companies that this can entail (Gowans, 2014).

Food banks and food banking can be understood as integral parts of the broader lived experience of poverty in the UK (Dowler and O'Connor, 2012; Purdam et al., 2016). Thus food poverty and the increased prominence of the food banking system within the welfare system can be seen as symptomatic of wider changes to public and welfare services, the expansion of the private sector, and the stripping away of employment protection and rights that underpin Wacquant's model of advanced urban marginality (Cummins, 2016). Receipt of emergency food aid is therefore an extreme manifestation of poverty and inequality (Garthwaite, 2016). The food banks who provide this aid are the primary response to food poverty on the ground (Wells and Caraher, 2014) and, as such, are spaces of practical and emotional support (Cloke et al., 2016) where the intersecting needs and forms of deprivation that arise from advanced marginality are addressed at the community level. However, these spaces are not state-run or funded. They are charity and community organisations that are legitimised and connected to the state largely via a system of referral.

### 1.3 Food banks and referral: mediating access

As Lambie-Mumford explains, food banks were originally designed to be an emergency intervention, providing food in the short-term while recipients await support from (typically) state welfare. They are third sector organisations, where food is primarily donated by local people and organisations, stored locally and with local distribution networks to those in need. Access to food bank services is mediated through referral from front-line service providers such as GPs, nurses, social workers, Job Centre staff and Family Support Workers via food bank vouchers (Lambie-Mumford, 2013). This means that health and social care professionals are positioned as the primary gatekeepers to third sector provision. Potential food bank users must convince these gatekeepers of their need in order to receive a voucher from them. The referral voucher system – as one that attributes ‘genuine need’ to food bank users – has been framed as inherently moralistic and judgmental, feeding into broader societal discourses of the ‘deserving’ and ‘underserving’ poor (Garthwaite, 2016; Williams et al., 2016). The referral system itself positions food bank users as recipients, not consumers, and reinforces ‘neediness’ as a qualifying criterium via the sometimes significant lengths that individuals must go to obtain a referral, thus creating a fundamental issue of food access based on need rather than rights (Lambie-Mumford, 2017) (p.59).

Foodbanks themselves limit the amount of assistance available to food bank users internally by capping the amount of referral vouchers available to households over set periods of time. In Trussell Trust food banks, users can be given up to three food parcels over the duration of their emergency or over a 6 month period. Where more is needed, they have to make special arrangements with the food bank via a voucher holder (the referring health and social care professional). This is fundamental for sustaining food banking as an emergency intervention and provides a mechanism for holding agencies who are supposed to be helping the potential recipients in the longer term to account (Lambie-Mumford, 2013). Some independent food banks operate similar capping policies, whilst others accept ‘self-referrals’ or give out food parcels without referrals (Citizens Advice, 2018). In some cases, rather than restrict access, some referral agencies use the food bank system to ease the burden on their own services (King's Fund, 2016).

While the experiences of food bank users have been explored qualitatively (Cloke et al., 2016; Garthwaite, 2016; Garthwaite et al., 2015; Williams et al., 2016) and referenced in calls for a Right to Food approach (Dowler and O'Connor, 2012; Lambie-Mumford, 2017), the perceptions of the health and social care professionals who refer to food banks and support the individuals using them remain underexplored. Ethnographic research on UK food banks has revealed that the progressive bleeding of welfare responsibilities that have traditionally been the remit of the state into the charity sector is an area of great concern for food aid organisations and the people they serve (Garthwaite, 2016). The experiences of those state employees working at the boundaries of this blurring between state and charity also warrant attention as the role they play in the administration of poverty and welfare is expanding in order to help negate the potential harms to health of austerity (BMA, 2017). Exploring the health and wellbeing challenges of contemporary food poverty therefore necessitates investigating the perspectives of multiple stakeholders in the food banking system. Specifically, by examining the accounts of those experiencing food poverty, the health and social care professionals who treat and refer them, the food bank organising community, and local authority teams tasked with addressing the logistic and public health implications of local food poverty. This paper reports on the findings of a qualitative study of the health and wellbeing challenges of food poverty and food banking and addresses the following research questions. First, what are the health and wellbeing challenges encountered by those experiencing or working with food poverty? Second, how do healthcare professionals and food aid organisations respond to these challenges? Finally, how does food poverty and food banking figure in broader narratives of marginalisation and exclusion?

## 2 Methods

The findings presented here are drawn from a larger qualitative study in Greater London comprising of two main elements: (i) an ethnographic investigation of the food bank system, staff and users, and (ii) a series of interviews with healthcare stakeholders. A total of 42 participants were interviewed. All fieldwork was conducted by the first author.

### 2.1 Data collection: Ethnographic study

This component explored the perspectives of food bank workers and users and investigated how the food banking system intersects with state agencies. Observations at food banks and Local Authority meetings were carried out alongside semi-structured interviews with food bank volunteers, organisers and users. For food bank observations, a mixture of activities including assembling food parcels, distributing food parcels, interactions between volunteers and food banks users, and team meetings were observed at four independent food banks spread over five visits totalling over 9 h. The food banks varied considerably. Two were reasonably large with more than 12 volunteers each and operated a referral voucher system closely emulating that of the Trussell Trust. Both of these organisations routinely collected data on food bank users and reported that families with young children made up the largest proportion of their clients. Although, anecdotally, volunteers from all of the food banks stated that recently there had been an increase in single-men seeking help. The remaining two food banks were much smaller and had evolved organically as the ‘food sections’ of charity organisations mainly offering non-food related support. These smaller food banks operated on an ad hoc basis – opening when they were aware of a spike in local need or when they could co-ordinate enough volunteers. They also operated on a voucher system, but in a much less structured way, taking internal referrals from other sections of their respective organisations.

In all cases, permission was sought to observe from food bank organisers and, when relevant, [first author] was ‘introduced’ to the food bank team as a researcher. On occasions when food bank users were present, [first author] was ‘chaperoned’ by a food bank volunteer and introduced to users as a researcher.

For the Local Authority observations (which included both local government staff, NHS employees and local food bank organisers), a range of group meetings including Food Poverty Working Groups, Community Nutrition Group Network meetings, (school) Holiday Hunger working groups, Food Partnership meetings and Food Poverty Action meetings were attended. A total of nine separate observations were carried out at meetings of these groups totalling over 20 h. As with the food banks, permission was sought to observe from the Chair (where relevant) and group members. Permission was sought to take notes during these meetings and, in some cases, [first author] was asked to offer an opinion on matters being discussed and sometimes invited back at a later date to present findings. The observations, at both food banks and Local Authority meetings, served as a way of accessing key local debates, framings, norms, issues and barriers associated with responses to food poverty. The perceptions and topics encountered in these observations were subsequently used to inform interviews and to compare how issues around food poverty were framed across different community contexts.

Food bank workers were interviewed once about their experiences of dealing with people experiencing food poverty, the referral process, and the challenges they faced. Food bank clients were each interviewed at least twice. The first interview focused on how they came to experience food poverty and subsequent interviews focused on the health challenges and their household food practices. The decision to conduct two interviews with food bank users – one on how they first came to use a food bank and one specifically on food practices and health - was based on pilot work. Narratives of hardship leading to contact and even reliance of third sector services, such as food banks, were significant and complex stories for food bank users that typically covered a range of issues including benefit and housing problems, relationship breakdown, immigration status and other assorted crises. Very little of these narratives were actually about food itself and were, overwhelmingly, about poverty, insecurity and crisis. In this context, moving the interview on to explore specific issues and details around nutritional health and practices seemed conversationally trivial after discussing life-altering events. Discussing food practices and health at a separate and subsequent interview proved much more conducive to focusing on topics related to food and nutrition.

Whilst initial meetings were typically in a food bank, subsequent interviews were mostly conducted in cafés, coffee bars and sometimes supermarkets (whilst participants shopped for food). Conducting interviews in these food-based settings was intended to help direct the interview towards the topics of food, eating and dietary health, by using place as a conversational prompt (Evans and Jones, 2011). The practice of interviewing food bank users across a range of spaces was largely participant led and partly pragmatic. Interviewing over a coffee and/or light refreshments in a café helped both to focus on food related issues and facilitate a more comfortable and conversational setting. Some of the participants, who gave very detailed and reflective accounts of their altered food practices, were happy to be interviewed whilst food shopping, so that they could expand on and explain some of the strategies they had mentioned in their previous interview. We took an emergent approach to engaging in such interviews, making the participants aware that is was an option if they thought it would be appropriate and comfortable for them. In all cases, we did not interview participants in their homes. Pilot work and discussions with community gatekeepers revealed that housing crises and problems at home were frequently a source of anxiety for those using food banks and so interviewing in (semi) public spaces convenient to the participants was a more appropriate approach.

### 2.2 Data collection: Healthcare stakeholder interviews

A series of semi-structured interviews were conducted with 20 London-based food aid-referring healthcare professionals. Participants were asked to explain how they first came into contact with food banks and comment on their interactions with those experiencing food poverty. Participants were also asked to comment on the public health implications of food poverty.

### 2.3 Recruitment and sampling

Food bank volunteers and organisers were recruited from the food banks visited as part of the ethnographic observations (described above) via snowball sampling, which started with an introduction from a colleague who volunteered at a food bank. Further, food bank staff were recruited from observations at local authority Food Poverty Working Group meetings and food bank observations themselves. Subsequently, these participants acted as gatekeepers to recruit food bank clients. Initially, this meant talking to volunteers who first came into contact with food banks as clients. They then provided introductions to people currently using food banks. A total of 8 food bank staff (volunteers and organisers) and 14 client families were recruited in this way. The sample was mixed in terms of gender, ethnicity, age, parental and immigration status.

Healthcare stakeholders were initially recruited by snowball sampling via introductions from clinical colleagues. Subsequent participants were recruited from local authority Food Poverty Working Group meetings and strategy meetings. Twenty professionals were recruited in this way and fell into three broad categories:

(i) **Healthcare providers (n** = **5)** (comprised of three General Practitioners, one Medical Secretary, and a Health Visitor)
(ii) **Local Authority health and wellbeing workers (n** = **9)** (comprised of three Early Years Dieticians, one Public Health and Wellbeing Team Specialist, one Family Support Worker, one Therapeutic Counsellor, one Public Health Training Officer, one Healthy Schools Programme Lead, and one Director of a regional authority food programme).
(iii) **Community and/or third sector health and wellbeing workers (n** = **6)** (comprised of one Weight Management Nutritionist for a social enterprise, two Community Engagement Managers for refugee charities, one Health and Wellbeing Project Co-ordinator for a homelessness charity, one Regional Food Programme Manager for a national advocacy group, and one Community Centre Outreach Administrator).

### 2.4 Ethics and informed consent

Full ethical approval was obtained from the anonymous Research Ethics Committee. All participants were given an information sheet, a consent form, and a verbal explanation of the study, which included information about what would happen to their data and their right to withdraw. Informed consent was obtained via consent forms, information sheets, and verbal explanations.

### 2.5 Data analysis

Interviews were transcribed verbatim and fieldnotes from observations were written up. All documents were then imported into NVivo10 and subject to a Critical Grounded Theory (CGT) analysis, which incorporates critical aspects of participatory philosophy into constructivist grounded theory in order to generate context-specific theory in settings with inherent social action agendas (Hense and Skewes McFerran, 2016). The construction and investigation of food poverty as a social problem has an inherent social action agenda. It infers a focus on structural inequality and inequities, rather than pathologising individuals, that is congruent with a rights based approach to the topic (Dowler and O'Connor, 2012) and the situating of lived experiences of food poverty within a framework of advanced marginality (Wacquant, 2007). In which case, ‘pre-concepts’ (values) around social justice and rights are employed in both the field and the analysis, meaning that a critical positionality must be adopted at the outset (MacDonald, 2001). In this sense, the approach to data collection is not wholly emic, it is situated within a research agenda and a body of literature that seeks to challenge the phenomena under study. Employing a critical grounded theory approach, therefore, is an acknowledgement of the situated nature of the undertaking and the implied positionality.

The following stages of data analysis were adapted from Charmaz's constructivist grounded theory (Charmaz, 2006) and Belfrage and Hauf's interpretation of CGT, with a distinct focus on the health challenges of food poverty as the identified ‘social problem’ (Belfrage and Hauf, 2017):

1. Open coding to conceptualise all incidents in the data.
2. Selective coding with health and wellbeing concerns as the tentative core.
3. Development of initial conceptualisations based on themes emergent from the selective coding.
4. Refinement of initialised conceptualisations based upon cycles of data collection and deskwork.
5. Development of a grounded conceptualization to explain the social problem at a given point in time.

In this approach, deployment and dissemination of the emergent theories are underscored by an awareness that theoretical saturation cannot be fully achieved. Critical grounded theories are therefore always provisional, incomplete and subject to revision, as they seek to characterise on-going, fluid and situated social problems and inequities (Belfrage and Hauf, 2017).

## 3 Results

The critical grounded theory that emerged from our analysis is that contemporary lived experiences of food poverty are embedded in, and symptomatic of, the perpetual uncertainty associated with precarious incomes, insecure housing and limited agency over other external factors. Food poverty was perceived to impact upon health and wellbeing both directly - in terms of dietary health - and indirectly through contributing to the amplification and perpetuation of marginalisation. These impacts are described in the sections below.

### 3.1 Barriers to providing adequate nutrition for babies and young children

One of the areas of greatest concern for those working with families experiencing food poverty was the impact on the health and wellbeing of children. Health and social care professionals explained how financial hardship and instability, which drive the emergence of food poverty, makes activities like meal planning, food-preparation and facilitating family meal times extremely difficult. Extended periods of financial hardship and uncertainty meant that these activities were deprioritised and occasionally abandoned altogether, especially when parents were in a perpetual state of crisis. Strategies of short-termism and survival in the context of eviction threats, uncertain incomes and family breakdown overshadowed many aspects of self-care and rendered long term health-related considerations such as diet to seem unimportant.

Dieticians and nutritionists, especially, consistently described food poverty as a barrier to breastfeeding. This is perhaps unsurprising given that women living on low incomes are the least likely to breastfeed and most likely to have the worst health and social outcomes for themselves and their children (Oakley et al., 2013) and the healthcare professionals interviewed were typically working in relatively deprived areas. As one Early Years Dietician explained, breastfeeding is *‘the healthiest and the cheapest option to feed a baby if you're on low income.’* However, this framing of the issue and concern over breastfeeding in particular was not shared by all participants. Food bank organisers described how families with children under a year were routinely offered infant formula milk, which can be helpful in times of crisis, and pointed out that substandard accommodation can be a barrier to safe bottle feeding in cases of extreme hardship through lack of access to clean water, refrigerators and storage space. Some healthcare providers and local authority workers explained that providing formula milk in a food bank can have the unintended consequence of helping to perpetuate the problem of low uptake of breastfeeding and that food aid settings were not the optimal context to make decisions about infant nutrition or to get advice about using formula milk.

By contrast, the food bank clients themselves did not appear to rank breastfeeding issues as the most pressing problem facing those raising young children in the context of food poverty. Those food bank clients who had been either pregnant and/or looking after young babies when experiencing hardship talked about struggling with depression and anxiety around trying to provide for them in such challenging circumstances. Having enough money to buy nappies (diapers) or finding a food bank that had them in the appropriate size was considered a much more pressing need. As Keina, a mother of two young children commented:

*The necessary things for mothers and especially single mums that don't have things … I will need to be begging somebody for my baby's diaper.*

While some participants engaged with, and enjoyed, the parenting support on offer in their local food banks, others did not welcome advice on how to feed their babies in this context. For example, Yaema, who experienced a range of crises when pregnant that meant she had to rely on a number of third sector organisations, was generally very positive about the services she received. However, she felt that being ‘interviewed’ about her parenting was too much.

*it's easier to advise somebody for what they want to do … but start saying you don't have to do this, it looks as if there's no freedom anymore, it looks as if you're controlling. And for the food banks, probably some of them should just stop prying into people's privacy, you know, if they've got referral to come and meet you, I'm sure the person that give them referral would have interviewed them, okay, you have this form and you go, we understand.*

Tensions between different actors in the food bank system around such issues were, to an extent, unavoidable. Referring health and social care professionals were focused on improving health behaviours and outcomes. Whereas food banks, although concerned about health, had a broader goal of trying to ease hardship. The agency, and even dignity, of some food banks users was sometimes obscured by these overlapping and often uncoordinated attempts to intervene by different agencies that has come to characterise the unclear placement of responsibilities for social welfare. Social and welfare policy in the UK is increasingly driven by risk management (Cummins, 2016) and the multiple agencies that food bank users encounter (both state and third sector) all have a duty of care, in this respect, to try and ensure that no unnecessary risk or unforeseen harms arise from their interactions with clients. The lived experience of these interactions, for those who are handled by such agencies, can be stigmatising and serve to accentuate the problems marginalized groups have to deal with (Wacquant, 2007).

### 3.2 Managing without fresh food, food storage and cooking facilities

Lived experiences of food poverty go beyond a lack of availability and affordability of nutritious food. The low incomes and insecure housing that drive people into food poverty also compromise food bank users’ ability to engage in healthy food practices. Participants explained that food bank use was often associated with housing crises, eviction and rent arrears, and came with the associated problems of staying in temporary accommodation such as inadequate access to cooking and food storage facilities. Stays in temporary accommodation and bed and breakfast can be very long in London, stretching into years (Aldridge et al., 2015). Families experiencing food poverty sometimes simply did not have access to adequate facilities to store and cook food for extended periods of time, which negatively impacts on dietary practices.

Local authorities and food banks are aware of this and have developed innovative ways of responding to the problems associated with living in temporary accommodation. Some food banks put together food parcels specifically for people with restricted food facilities. Cooking classes and ‘try a new food’ activities delivered through food banks were some of the ways in which poor dietary health were addressed. Some food banks worked with nutritionists to ensure that food parcels were as healthy as possible, developing their own tailored nutritional guidelines for food parcel contents and educating volunteers and clients at the same time. There was a conscious attempt to foster a positive and healthy food environment in some food banks, which added to a sense of community amongst both volunteers and clients. Alan, the organiser of a very active food bank, explained how they had several nutritionists on their steering group, ran cooking classes and had people on-site to advise clients on nutrition issues:

*Yeah, she's just joined as our latest nutritionist but we've had others …. [Local Authority] Nutrition Partnership, who were able to come along on the night and help the visitors understand how they can make a tasty meal with products, fresh products particularly, that they would normally not know what to do with.*

Cooking classes for those of low incomes and skills-based interventions delivered in food aid settings have been criticised for contributing to neo-liberal framings of food poverty as an issue of individual failure or something that can be tackled via education rather than redistribution (Caplan, 2016). Within an advanced marginality framing, this would be interpreted as the state and third sector being implicated in a political agenda that recasts the problem of (food) poverty and deprivation as the outcome of individual lifestyle choices and can therefore be subsequently used to justify punitive trends and structural stigmatisation in social policy (Cummins, 2016).

However, like the food bank organiser quoted above, the food bank users interviewed for this study also spoke highly of the food bank cooking classes they had attended and reported enjoying them. Health and social care professionals offered a slightly different slant on the issue, with one Dietician pointing out that, like many of the cooking classes run by Local Authorities, *‘they are a way of getting food into people’*. Both food bank and Local Authority cooking classes provided all the ingredients, and many included time for the attendees to eat a shared meal together at the end of the session and take home leftovers. In this sense, cooking classes themselves can be thought of as secondary food aid, which may go some way to explaining their popularity as they provide a way of receiving food without having to declare ‘need’ and incur any of the associated stigma.

While the food bank model provides relief and alleviation for the ‘symptoms’ of food poverty through food parcels and cooking classes, it does not tackle its root causes. Food banks can only work with the food, donations and support they receive as they are not state-funded. While larger, better supported and organised food banks can achieve more, smaller, independent and ‘unofficial’ organisations have significantly less capacity to promote dietary health. The non-perishable food-stuffs that are routinely donated cannot always constitute a nutritious diet. This can make maintaining a healthy diet from donated food challenging (Garthwaite et al., 2015; van der Horst et al., 2014). Yaema (quoted above) also explained that feeding her young children with donated food could be challenging as it left her unable to cater to her own and her children's tastes and cultural preferences.

*Sometimes you just limit your choice of cooking because of what you get and it takes time to persuade the kids to have it, because I'm African and most time we get used to eating our food and when you go to the food bank it's not what you want to get but you just have to get used to eating it.*

In some cases, a lack of culturally appropriate food resulted in food waste. Food banks are aware of these problems and, when possible, give clients the option of the more common cultural diet restrictions such as no pork, no beef, vegetarian and (sometimes) Halal. However, ‘culturally appropriate’ can also be taken to mean familiar foods. Some ranges of processed and tinned foods are unfamiliar and unpalatable to food banks users who have not grown up with them. Keina (also quoted above), a recent migrant to the UK, explained this problem

*Like me they give me soups. If you go to my kitchen wardrobe [sic] now because I don't want to throw food away, I'll see soup, you see soup there in my cupboard that I don't take. And I don't want to throw them away …. I don't want to waste it.*

Keina loathed throwing away food, as she explained being very grateful for it. But tinned soup (a very popular donation to food banks) was not something that she or her children ate. In a similar vein, Linda, a working single mother, explained that relying on food bank parcels meant that she could afford to pay the rent. But it also meant going without fresh food.

*It does have an impact on health, because you don't get anything fresh, I mean that's just the nature of it, everything's packaged in packets and jars and tins, and obviously it's people that have donated things … I had a mugshot [packet soup in a cup] thing and noodles in a packet thing, you know, just really, I wouldn't have fed it to my daughter really to be honest, I would have rather gone and stolen food from a shop, but I was taking it in for lunch and they were like, “Oh my god, I'm so surprised that you eat stuff like that,” I said, “This is the stuff you get when you go to a food bank.” You know, this is it.*

Linda went on to describe a practice commonly reported among the parents interviewed; that of putting aside the best quality and most nutritious food from the donated parcel for her child and having whatever was left over for herself. This is congruent with the notion of parents, and particularly mothers, acting as ‘shock absorbers’ (S. Hall et al., 2013) for the worse effects of poverty on their children. In the UK, austerity driven welfare reform has had a gendered impact of familial relations, with expectations of financial, social and economic ‘sacrifices’ disproportionately ascribed to women, and particularly to mothers. This, in turn, has implications for the health and wellbeing of these women (S. M. Hall, 2016).

### 3.3 Exacerbating health and social problems

Experiencing food poverty can create new health and social problems and worsen existing ones. Participants experiencing food poverty explained that issues such as stress, depression and weight-gain were made worse by these experiences and more difficult to manage. Trying to adhere to a restricted or specialised diet for medical reasons was especially difficult. However, the ways in which food poverty can worsen existing problems can be more fundamental than dietary problems and are near impossible to anticipate and plan for. This is aptly demonstrated in Trevor's account, below, who has not been in regular paid employment for more than ten years, as a result of a variety of health problems. He had to use food banks for an extended period when his benefits were sanctioned.

*Trevor: They stopped, they stopped my money.*

*CT: Who stopped your money?*

*Trevor*: *DWP and then I had to appeal against it to get it back. What was it, about 6 months weren't it … Like, I walk all the way down the food bank and then try and carry it all the way back. Took me three quarters of an hour to get down there and then took me about two and a half hours to get home because I suffer with COPD and every so often I have to use my pump.*

Without funds for food or transport, Trevor had no other choice but to walk to the food bank and carry his food back home. Due to the nature of food parcel contents – often jars, tins and boxes of non-perishables – this meant a very long walk home with heavy shopping which was physically difficult and which aggravated his COPD. Trevor lived alone and so had no one to help with the trip. Severe hardship resulting in food bank users having to walk considerable distances to obtain food is a well-documented issue (Garthwaite, 2016). Situating this phenomenon in the context of overlapping vulnerabilities illuminates the multiple ways in which it can impact upon health.

This conflict of competing vulnerabilities and risk was also commented upon by healthcare professionals. Helping patients struggling with such issues is not straightforward. Household interaction with food aid typically occurs during other episodes of health crisis, which prompt them to turn to healthcare professionals. Food poverty is rarely the ‘presenting problem’ and is often embedded within a suite of other complaints. Frontline healthcare professionals have to deconstruct complex accounts of poverty and find ways to intervene. Japp, a GP working in a deprived London borough explained the inherent difficulties of treating patients experiencing extreme hardship, in which food poverty and health complaints are often part of a constellation of stressors and problems that need to be unpicked during the allotted 10-minute consultation:

*Because as you can imagine, if you're marginalised, you are unreliable, you have loads and loads and loads of problems, so in 10 minutes it's impossible, it's stupid, impossible. So either the doctor gets really defensive or offensive and fobs the person off, or you do something which fizzles out in nowhere … … it's (food poverty) not a discreet entity, it's part of all the other bits of [not] having fuel, clothes, access to information …. …. but in a health encounter, if the focus is by default not on poverty, the focus by default is on medicines and complaints and the system handles it like this, so it's difficult if a person comes with a health complaint to ignore that and to move away to poverty.*

As Japp explains, a 10-minute consultation is inadequate to deal with the complex and interrelated health and social problems of extreme marginalisation in which food poverty occurs. If the GP focuses on the presenting health complaint they risk doing so at the expense of delving into issues of poverty and deprivation that underscore them and vice versa. There is simply not enough time or resources to develop and explore ways to help with the linked problems of deprivation *and* health. Growing pressures and responsibilities for healthcare professionals dealing with vulnerable patients has been identified as one of the biggest challenges facing general practice (King's Fund, 2016). Such pressures increase workloads, put strain on the doctor-patient relationship, takes up appointment spaces, and disproportionally impacts on the delivery of services in deprived areas where need is greater (Matthews-King, 2015). All of which are likely to negatively impact on the quality of care for the most vulnerable. In this way, significant groups of people are effectively locked out of access to resources (such as health and social care) (Cummins, 2016) that form the basis of citizenship and, thereby, contributes to extreme marginality and precarity (Wacquant, 2007).

Bill, a GP in a deprived London borough, recounted the story of one of his patients who, after suffering with mental health problems, long-term unemployment and morbid obesity for many years, finally had bariatric surgery to lose weight and subsequently found some part-time casual work with a local charity. However, the erratic and causal hours of work caused problems with the benefits system and she eventually lost her benefits and exemptions. Aside from the general hardship this caused, the accompanying loss of entitlement to free prescriptions meant that she could no longer afford to take all of her medications which, as a result of her surgery and ongoing mental health problems, were numerous. In order to try and deal with this she started prioritising her limited funds for what she viewed as her ‘most important’ medications and simply not taking the rest. Additionally, she eventually requested a referral to a food bank to save money for her prescription medications. However, as has been described above, constructing specialised and restricted diets (like that following bariatric surgery) from donated food is very challenging. Eventually, his patient came back to seem him in a distressed state and he signed her off of work, meaning that she was unable to continue in paid work and instead began to reclaim benefits in order to regularise her income and maintain her health. As Bill explains below.

*She was very you know upset … had been going to the food bank to get sort of a limited amount of food, couldn't get any of her prescriptions … … so she's back out of work and now she gets sort of free prescriptions so she's able to have her medication … I think it will be very difficult for her to think again about being able to sort of go back into employment having had such a disastrous time … She'd got a sort of long course of treatment with our psychology team … to help her get back to where she was, so you know I think it'll be difficult for her to get back there basically.*

Her existing medical issues acted as an additional layer of vulnerability that left Bill's patient unable to weather the combination of precarious employment and inflexible welfare benefit administration that her trying to get back into paid work entailed. In this instance, the potential health cost of food poverty removed her from the labour market.

## 4 Conclusion

This study has revealed that the health and wellbeing challenges encountered by those experiencing food poverty are both direct and indirect. Direct problems largely related to the challenge of maintaining a good diet and the impacts of prolonged periods with a restricted choice of foods, especially for young families. Narratives around indirect health and wellbeing challenges are centred on the worsening of existing health conditions and an amplification of personal vulnerability. Lived experiences of food poverty and food banking are both embedded in extreme precarity and marginalisation and also serve to perpetuate them. Uncertainty, hardship and lack of agency around food were part of broader precarious experiences of the social determinants of health, most notably income and housing. This suite of experiences can be understood as a form of ‘advanced marginality’, as a combination of extreme economic, political, social and cultural exclusion that produces poverty not as a residual category, but as the consequence of a changing wage-labour and welfare relationship (Wacquant, 2007).

This paper explored food poverty and the food banking system from multiple perspectives, moving beyond a specific focus on food banks and exploring wider elements of the welfare system including Local Authority infrastructure and local health and social care professionals that both rely on and help to embed the food bank system in the UK welfare landscape. Food banks start where the welfare safety-net stops, with health and social care professionals in the position of having facilitate this transition (Matthews-King, 2015). Examining the processes through which this occurs is key to understanding the local mechanisms of state retreat and the subsequent increasing role of the third sector in shouldering the burden of social welfare provision.
